# Does Google™ Have the Answers? The Internet-based Information on Pelvic and Acetabular Fractures

**DOI:** 10.7759/cureus.5952

**Published:** 2019-10-21

**Authors:** Evelyn P Murphy, Christopher Fenelon, Fiona Murphy, MN Baig, Robert P Murphy, Megan Diack, Michael Leonard

**Affiliations:** 1 Orthopaedics, Cappagh National Orthopaedic Hospital, Dublin, IRL; 2 Orthopaedics and Trauma, University Hospital Galway, Galway, IRL; 3 Orthopaedics, University Hospital Galway, Galway, IRL; 4 Internal Medicine, University Hospital Galway, Galway, IRL; 5 Orthopaedics and Trauma, Tallaght University Hospital, Tallaght, IRL

**Keywords:** readability, reliability, internet-based information

## Abstract

Introduction

The aim of this study is to assess the readability and reliability of internet-based information on pelvic and acetabular fractures.

Methods

The three most popular English-based internet search engines are Google, Yahoo, and Bing. Quality was assessed using the DISCERN tool, the Journal of the American Medical Association tool, and the presence of the Health on the Net Code (HONcode) seal. Readability was assessed using a combination of the Flesch Reading Ease Score and the Flesch-Kincaid grade level. Inclusion criteria included English language websites with the relevant search terms. We excluded videos, YouTube links, or sponsored advertisements. Search terms included acetabular fracture/fractured acetabulum and pelvic fracture/fractured pelvis. The top 25 websites in each search engine were reviewed. The searches for acetabular fractures and pelvic fractures generated 75 websites in total. Duplicates were excluded.

Results

The search for acetabular fracture revealed 36 discrete websites among the three search engines, and the search for pelvic fractures revealed 45 websites. Overall, the average reading grade was 9.7 for acetabular websites and 13.6 for pelvis websites. The quality of the websites was poor across all key performance indicators studied.

Conclusion

Physicians should be aware of the quality of medical information available to patients via internet searches because physicians should play a central role in the navigation of poor quality information to help direct patient-centered care.

## Introduction

Increasingly, patients are using the internet to complement their existing knowledge of a condition. An estimated 61% percent of people would conduct online research into a topic once given a diagnosis [1]. It is important that the information accessed is relevant, reliable, and trustworthy. Also, the medical community is becoming reliant on the internet as a source of trusted information. Among physicians, 63% report conducting online research and changing an initial diagnosis based on their findings [1]. Common search engines, like Google or Yahoo, are used by 46% of physicians as a frequent information source [2]. Also, 53% of physicians believe that one of the main barriers to communication is misinformed patients [1]. Nearly 90% of physicians reported that improved access to online medical information had improved the quality of care they provided.

Medicine has evolved from a didactic model of care towards a partnership whereby patients expect to be involved in the delivery of care. Pelvic and acetabular (P&A) injuries have the potential to confer significant morbidity to patients. The nuances of online information can influence a patient’s interpretation of information. Thus, it is incumbent upon physicians providing P&A care to have a degree of insight into the challenges and potential misconceptions that exist. This will enable the surgeon to direct patients appropriately or even dispel some falsehoods.

## Materials and methods

**Figure 1:**
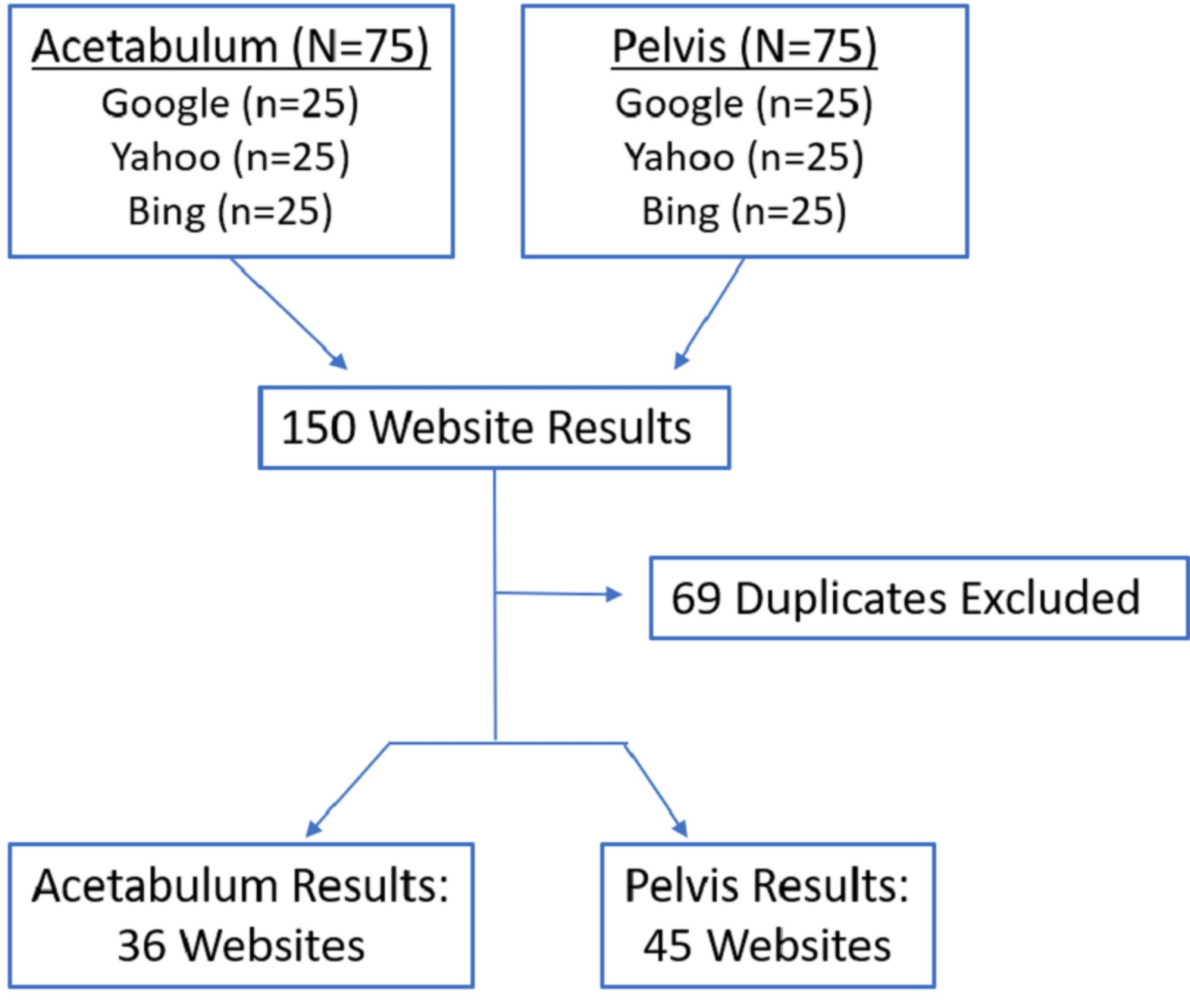
Website Searches

Inclusion criteria included English language websites with the relevant search terms. We excluded videos, YouTube links, or sponsored advertisements. Search terms included acetabular fracture/fractured acetabulum and pelvic fracture/fractured pelvis. The top 25 websites in each search engine were reviewed. The searches for acetabular fractures and pelvic fractures generated 75 websites in total. Duplicates were excluded. An overview of the study methodology is presented in Figure 1. The search terms used for this study are presented in Table 1.

**Table 1:** Search Terms

Acetabular search terms:	Pelvic trauma search terms
Acetabulum fracture	Pelvic fracture
Fractured acetabulum	Fractured pelvis
Broken socket	Broken pelvis
Fractured socket	

Website grading and assessment

The websites were assigned grades based upon their affiliations. This consisted of the following categories: personal, academic/affiliated with a university, physician maintained, non-profit, or commercial.

An online free calculator was used to generate the readability scores [3]. Readability was defined as a measure of the reading skills an individual must have to understand the material. The information was tested for readability using the Flesch Reading Ease Score (FRES) [4] and the Flesch-Kincaid Grade Level (FKGL) [5], both of which are validated assessment scores. The FRES uses a scale of 0 to 100, with lower scores correlating to increased difficulty in readability. The FKGL score uses a formula to determine the reading grade in the United States (US). The FKGL score is inversely proportional to the FRES score.

The information was assessed for reliability using the presence of a Health on the Net Code (HONcode) certification or seal, the DISCERN tool, and the Journal of the American Medical Association (JAMA) benchmarks. A HONcode certificate is provided to websites that meet Health on the Net standards [6]. The DISCERN tool is a questionnaire with 16 separate domains, each scored on a scale of one to five [7]. It allows one to evaluate the information being provided in a standardized fashion. The JAMA established four key criteria for websites consisting of the presence of references, authorship, presence of disclosures, and evidence of information being updated [8].

Data analysis

Two independent orthopedic registrars were responsible for data abstraction. The search terms as described were entered into our records. Results were stored on a Microsoft® Excel spreadsheet (Microsoft® Corp., Redmond, WA) and analyzed using the IBM Statistical Package for Social Sciences (SPSS) for Windows, Version 24.0 (IBM Corp., Armonk, NY). Data are presented as mean, percentage, and standard deviation (SD), if appropriate. Statistical significance was calculated using the student paired t-test (P < 0.05).

## Results

The search terms returned 171,447,500 websites across three search engines (Google, Bing, and Yahoo). We filtered these results by keyword search filters according to language, orthopedics, surgery, acetabular, and pelvis to arrive at 81 relevant websites. There is an abundance of information on P&A trauma on the internet. However, the quality of such information is dubious.

The mean FRES score for acetabular internet information was 43.83 ± 23.04, while the mean FRES score for pelvic internet information was 41.38 ± 17.62. The mean FLKG for acetabular information was 9.68 ± 5.34, while the mean FLKG for the pelvis was 13.57 ± 6.4. Of the 81 websites analyzed, only 7.4% (6/81) had a reading grade of equal to or lower than a sixth-grade level.

The JAMA benchmark is made up of four criteria to assess the quality of information [8]. The scale runs from zero to four, and it includes names of authors, references, currency/updating information, and disclosures. The average JAMA score for the acetabular information was 2.17 ± 1.48. The average JAMA score for the pelvic information was 1.68 ± 1.48. Disclosures were the criterion most frequently absent in both data sets, followed by references. Table 2 demonstrates the adherence to the JAMA criteria.

**Table 2:** Journal of the American Medical Association (JAMA) Criteria

	Acetabular websites	Pelvis websites
Author	24 (68.5%)	25 (54%)
Currency	22 (62.8%)	22 (47.8%)
References	18 (51.4%)	19 (41%)
Disclosures	12 (34.2%)	10 (21%)

The HONcode was only present in one of the 36 acetabular websites (2.8%) and two of the pelvic websites (4.4%).

The DISCERN tool was then used to determine the mean scores for both data sets. According to the DISCERN analysis, the mean acetabular score was 32.4 ± 11.7; the mean pelvic score was 33.64 ± 13.8. The scale runs from 16 to 80. The higher the score, the higher the quality of the information. Thus, all data performed poorly upon interrogation.

Website analysis

The websites were categorized into five subtypes. Table 3 shows the types of websites available for review. Commercial websites comprise 19.4% of acetabular websites and 31.1% of pelvic websites. In terms of transparency and providing updated information, the sites we analyzed scored poorly. Less than 5% of the websites had the HONcode. Most of the websites (80%) lacked disclosures for pelvic trauma.

**Table 3:** Website Categories

Categorization	Acetabular websites N = 36	Pelvic websites N = 45
Personal maintained	3	0
Academic/University-affiliated	20	12
Physician maintained	4	8
Nonprofit/public health	2	12
Commercial	7	14

## Discussion

P&A fractures are bimodal in distribution. They tend to affect younger patients after high-energy trauma and elderly patients with low impact trauma. While P&A fractures make up only a small number of all fractures, they are often associated with significant morbidity. Approximately 26% of patients experience posttraumatic arthritis and complication rates up to 45% [9]. The understanding and management of these injuries are often poorly understood by patients.

Research has shown that 47% of the global population (3.2 billion people) use the internet and have access to an expanse of information [10]. This level of access is partly made possible by the explosion of smartphone use, with 80% of the adult population expected to own an internet-connected smartphone by 2020 [11]. However, the reliability and readability of this information are often very poor, as a number of medical studies have highlighted [3-4]. P&A injuries are often treated at specialist centers. Patients can experience a staged approach to their surgeries, which can lead to ample time and opportunity to investigate symptoms on the internet.

A survey of 1,014 adults in the United Kingdom revealed that 61% of all adults use technology to access some form of healthcare services [10]. A report by the Pew Research Center’s Internet and American Life Project [12] commissioned in 2013 in the US found that one in three adults use the internet as a diagnostic tool. For 46% of those surveyed, what they read online led them to seek medical expertise [12]. When they did, 41% had confirmation of suspicions.

The quality of much of the information available via the internet is poor; however, online research will only grow. The model of care provided towards patients has moved from the traditional didactic model of ‘doctor knows best’ to a shared decision-making process that encourages patient engagement. Well-informed patients can express personal values and opinions.

A criticism of utilizing the internet as a sole source of information is the lack of clarity and the questionable quality of the information which is accessed. Trust in the internet is often debated; however, it is a matter of attrition and familiarity, as more patients and physicians possess smartphones with access to the internet. Convenience is a well-recognized feature of the internet [13]. A particular concern of the internet information on P&A fractures was the high prevalence of commercial websites. These websites have an inherent bias, which can misdirect care. The average reading grade age for P&A information available online is beyond what is recommended for an average population. In terms of transparency and currency, these websites failed dramatically. Fewer than 5% of the websites had the HONcode certification. A worrying 80% of websites lacked disclosures for pelvic trauma. An overall lack of references, disclosures, currency, and author credits are a worrying trend among the top websites included in this study. A report from the Pew Center found that while patients do go online, the primary health care provider remains integral to the navigation and interpretation of information online [13].

## Conclusions

While physicians maintain a central role in providing care to patients, patients are using the internet as an additional information source. Unfortunately, the information being accessed is of poor quality in terms of readability and reliability. Centers that provide specialist care for P&A trauma should endeavor to aid patients in the navigation of such information.
